# Comparison of health-related quality of life among men with different co-existing severe mental disorders in treatment for substance use

**DOI:** 10.1186/s12955-017-0781-y

**Published:** 2017-10-23

**Authors:** Ana Adan, Julia E. Marquez-Arrico, Gail Gilchrist

**Affiliations:** 1 Department of Clinical Psychology and Psychobiology, School of Psychology, University of Barcelona, Passeig de la Vall d’Hebron, 171, 08035 Barcelona, Spain; 2 Institute of Neuroscience, University of Barcelona, Passeig de la Vall d’Hebron, 171, 08035 Barcelona, Spain; 3 National Addiction Centre, Institute of Psychiatry, Psychology, and Neuroscience, King’s College London, 4 Windsor Walk, Denmark Hill, London, SE5 8BB UK

**Keywords:** Health-related quality of life, Substance use, Dual diagnosis, Schizophrenia, Bipolar disorder, Major depressive disorder, Comorbidity

## Abstract

**Background:**

Patient-perceived health-related quality of life has become an important outcome in health care as an indicator of treatment effectiveness and recovery for patients with substance use disorder. As no study has assessed health-related quality of life among male patients with substance use disorder and co-existing severe mental illness, we compared health-related quality of life among patients with substance use disorder and the following severe mental illness diagnosis in Barcelona, Spain: schizophrenia, bipolar disorder, major depressive disorder, and examined the associations with clinically related variables. Additionally, we compared results for health-related quality of life in patients with substance use disorder and severe mental illness, with Spanish population norms.

**Methods:**

We assessed 107 substance use disorder male patients using the 36-Item Short Form Health Survey comparing results across three groups with: comorbid schizophrenia (*n* = 37), comorbid bipolar disorder (*n* = 34), and comorbid major depressive disorder (*n* = 36). Multiple analyses of variance were performed to explore health-related quality of life by the type of co-existing SMI and linear regression analyses examined clinical correlates for the 36-Item Short Form Health Survey dimensions for each group.

**Results:**

There were differences in Physical Functioning, Vitality and the Physical Composite Scale among groups. Poorer Physical Functioning was observed for patients with comorbid schizophrenia (80.13±3.27) and major depressive disorder (81.97±3.11) compared with comorbid bipolar disorder patients (94.26±1.93). Patients with substance use disorder and schizophrenia presented lower scores in Vitality (41.6±2.80) than those with co-existing bipolar disorder (55.68±3.66) and major depressive disorder (53.63±2.92). Finally, results in the Physical Composite Scale showed lower scores for patients with comorbid schizophrenia (51.06±1.41) and major depressive disorder (51.99±1.87) than for those with bipolar disorder (60.40±2.17). Moreover, all groups had poorer health-related quality of life, especially Social Functioning, Role-Emotional and Mental Health, compared with population norms. Different clinical variables (e.g. medical disease comorbidity, severity of addiction, psychiatric symptomatology, suicide attempts, drug relapses) were related to different health-related quality of life dimensions depending on the co-existing severe mental illness.

**Conclusions:**

Among male patients with substance use disorder, co-existing severe mental illness may influence some health-related quality of life dimensions and clinically related variables. Such differences may require tailored therapeutic interventions.

## Background

Substance use disorders and severe mental illness commonly co-exist (65–85%) in patients seeking treatment for either condition [1, 2]. According to previous studies, the most common severe mental illnesses among patients with substance use disorder were schizophrenia, bipolar disorder and major depressive disorder [2]. These co-existing conditions are usually referred to as dual diagnosis [3]. Having a dual diagnosis is strongly associated with major clinical impairments including suicide attempts [3], severe clinical profiles and symptomatology [4], poorer prognosis [5], relapses in both disorders [6, 7] and poorer quality of life [8–10]. Therefore, treatment and recovery of patients with dual diagnosis is a challenge for both drug and mental health treatment providers. Several studies have explored the best therapeutic approaches and strategies to address these co-existing conditions and found that integrated long-term interventions, disorder specific cognitive-behavioral strategies and contingency management have better results [11, 12].

Treatment approaches considering health-related quality of life (HRQoL) are likely to be more effective in patients with substance use disorder, as they address social needs, adopt a chronic disease model of addiction and track symptom severity and distress levels [13, 14]. Patient-perceived HRQoL has become an important outcome in health care as an indicator of treatment effectiveness and recovery [15, 16]. While previous studies have compared HRQoL between people with dual diagnosis and substance use disorder and/or severe mental illness, no study has examined HRQoL among patients with dual diagnosis and different mental health disorders. Such data could be useful to inform possible targeted treatments for dual diagnosis by providing a subjective complementary perspective to clinicians, reporting unique information about patients’ needs and insights that could enhance the effectiveness of health care [17, 18].

Patients with substance use disorder and co-existing schizophrenia report poorer physical functioning, vitality and, in general, lower HRQoL than patients with substance use disorder [19, 20]. In addition, they report poorer social functioning and more emotional problems [10, 20] than patients with SZ. Patients with substance use disorder and co-existing bipolar disorder,present poorer physical and psychological health and more impairments in their social relationships than patients with bipolar disorder only, substance use disorder only,and healthy controls [21, 22]. Depressive symptoms [23], suicide attempts [24], illicit drug use [25] and medical disease comorbidity [23, 25] were also associated with a reduced HRQoL in patients with bipolar disorder. Moreover, HRQoL in patients with bipolar disorder is lower, even though they do not experience (hypo) manic or depressive episodes [26].

Regarding patients with substance use disorder and comorbid major depressive disorder, alcohol use was related to poorer physical health, psychological functioning, social relationships, and environmental factors [27], while general health perception and role-emotional were negatively correlated with depressive symptomatology [28, 29]. Although such findings contribute to an understanding of dually diagnosed patients, the possible differences among them depending on their severe mental illness remain unknown.

As HRQoL measures in patients can greatly assist clinicians to develop models that engage patients as partners in their own care [17], we examined for the first time, the possible differences in HRQoL in a sample of patients with substance use disorder and different comorbid severe mental illness (substance use disorder with co-existing: schizophrenia, bipolar disorder, and major depressive disorder), to determine associated factors. Moreover, we compared HRQoL for patients with substance use disorder according to their severe mental illness with Spanish population norms to guide clinicians about possible treatment needs related to comorbidity. This study provides data that encourages the adoption of an integrated recovery-oriented model that considers wider outcomes than abstinence as the main goal of treatment since poor quality of life is not only addiction-specific [12, 30].

## Methods

### Study design, participants and procedure

**Fig. 1:**
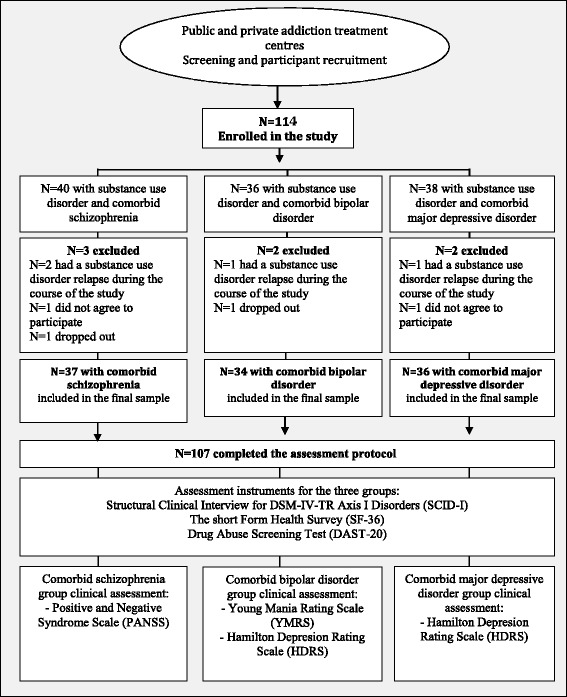
Flowchart of participant recruitment and assessment protocol

Participants were recruited from public and private substance use disorder treatment centers from Barcelona, Spain. Psychiatrists and psychologists informed potential eligible patients about the study and arranged a time for the researcher to discuss the study in detail before gaining their informed consent to participate. Those participants providing informed consent were included in the study if they were: (1) male; (2) aged between 18 to 55 years old; (3) had a current diagnosis of substance use disorder in remission for at least three months but were still receiving substance use disorder treatment; (4) reported no substance use disorder relapses for at least one month before participation in the study; (5) had a current diagnosis of schizophrenia, bipolar disorder or major depressive disorder; and (6) were able to complete instruments in Spanish. Only male participants were included as the majority of patients in treatment for substance use disorder are men [8], and the prevalence of severe mental illness in substance use disorder also tends to be higher in men than in women [2, 10]. The exclusion criteria were: (1) meeting DSM-IV-TR criteria for a current substance-induced psychiatric disorder or a psychiatric disorder due to a medical condition and (2) unstable or uncontrolled psychiatric symptomatology. Severe mental illness was diagnosed in all patients in our sample before participating in our study. This process ensured that patients were in a clinical stable condition to participate and that they did not present other symptoms not related to their psychiatric diagnosis of schizophrenia, bipolar disorder, or major depressive disorder. The sample comprised 107 male patients with a substance use disorder and comorbid severe mental illness, with a mean age of 39.78 years (*SD* = 8.11). Patients were not compensated for their participation in the research. These patients were all diagnosed with substance use disorder and co-existing: schizophrenia (*n* = 37), bipolar disorder (*n* = 34), or major depressive disorder (*n* = 36). A trained psychologist administered all clinical assessments, and patients completed the SF-36 Health Survey alone or with the help of the psychologist if required due to poor literacy skills. Figure 1 describes the assessment and recruitment of patients.

### Assessment instruments

#### Structural clinical interview for DSM-IV-TR Axis I disorders (SCID-I)

Current diagnoses of substance use disorder and severe mental illness were obtained by treatment providers of each respective participant and confirmed through administration of the SCID-I [31]. SCID-I and a clinical interview design for our study were used to assess sociodemographic (e.g. age, marital status, social class, schooling and economic status) and clinical variables (e.g. psychiatric and substance use family history, age of onset of the disorder and consumption, relapses, abstinence periods, type of drugs used, suicide attempts, presence of organic pathology and medication).

#### The short form 36 health survey (SF-36)

The SF-36 [32] is a 36-item measure of HRQoL and consists of eight primary components: Physical Functioning, Role-Physical, Role-Emotional, Social Functioning, Mental Health, General Health, Bodily Pain, and Vitality. Scores in the SF-36 range from 0 to 100, where a higher score indicates a better health related quality of life. The SF-36 includes an additional self-perceived item measuring changes in general health over the last year (Health Transition item). The scale provides two secondary composite standardized scales using T scores (with a mean of 50 and standard deviation of 10): the Physical Health Component Summary and the Mental Health Component Summary. The SF-36 Spanish version was used as it demonstrated good psychometric properties and reference population values [33]**.**

#### Drug abuse screening test (DAST-20)

The Spanish version of the DAST-20 [34] was used to assess severity of substance use disorder as it showed adequate psychometric properties [35]. The DAST-20 is composed of 20 questions and provides a total severity score from 0 to 20 (1–5 low, 6–10 intermediate, 11–15 substantial, 16–20 severe).

#### Positive and negative syndrome scale (PANSS)

Psychotic symptomatology was measured in the comorbid schizophrenia group using the Spanish version of the PANSS [36], which has been described as a reliable instrument [37]. The PANSS is a 30-item scale measures four areas related to different symptomatology: Positive Syndrome, Negative Syndrome, Composite Scale, and General Psychopathology. The Positive and Negative Syndrome direct scores ranges from 7 to 49, while the General Psychopathology ranges from 16 to 112. The Composite Scale shows the predominance of positive or negative symptoms and its direct score ranges from −42 to 42 (Positive minus Negative direct scores). All PANSS direct scores were transformed to percentiles according to Spanish normative data [37].

#### Young mania rating scale (YMRS)

The YMRS [38] was used to measure manic symptomatology in the comorbid bipolar disorder group. The Spanish Version of the YMRS is a useful, valid and reliable tool for the assessment of manic symptoms [39]. This instrument is an 11-item clinical scale designed to assess the severity of manic symptoms in several areas (e.g. elevated mood, increased motor activity/energy, sexual interest, sleep). Each item includes four explicitly defined levels of severity. Severity ratings are based on the patient’s subjective report of his clinical condition during the last 48 h and the clinician’s observations during the interview. The total score related to the severity of the affective state following these criteria: < 6 euthymic, 7–12 subclinical symptoms, 13–20 hippomanic and >20 manic episode.

#### Hamilton depression rating scale (HDRS)

The Spanish version of the 17-item version HDRS was used to measure depressive symptomatology in the comorbid bipolar disorder and major depressive disorder groups. This version showed good psychometric properties [40, 41]. The HDRS total score ranges from 0 to 53 and is interpreted as follows: 0–7 no depression, 8–13 low, 14–18 mild, 19–22 severe, and >23 very severe depressive symptoms [41].

### Statistical analysis

Descriptive statistics were used to describe the sociodemographic and clinical profile of the sample. Intergroup differences were explored by performing univariate analyses (ANOVA) for continuous data and Chi Square tests for categorical data. Multivariate Analyses of Covariance (MANCOVA) and post-hoc analyses were applied to explore differences in HRQoL dimensions among the three groups, with group as the fixed factor and age as a covariate as it could be a confounding factor [42]. Bonferroni correction was applied to address the problem of multiple comparisons and to protect against type I errors. Partial eta square was obtained as a measure of MANCOVA/ANOVA effect size (0.01 small; 0.06 medium; 0.14 large) as well as Cohen’s d (0.2 small; 0.5 medium; 0.8 large) for post-hoc contrasts [43]. Moreover, to obtain evidence about the degree of impairment in the HRQoL dimensions, T scores (mean = 50; *SD* = 10) were calculated for primary SF-36 dimensions of each group according to Spanish population norms [33].

Bivariate correlational analysis between clinical variables and SF-36 dimensions were performed to explore correlates of HRQoL in each group. Categorical variables were dummy coded for further analyses. Only variables that correlated with HRQoL in bivariate analyses and showed a ß standardized coefficient ≥ 0.4, were retained for the multiple stepwise regression analyses performed to determine factors related to HRQoL dimensions among the three groups. Data were analyzed using the IBM SPSS Statistics version 22 [44].

## Results

### Study sample characteristics

Tables 1 and 2 summarize the sociodemographic and clinical data for the three groups. Groups differed significantly by age (*p* = 0.012), years of schooling (*p* = 0.039), and economic situation (*p* = 0.005). Clinical variables, such as medical disease comorbidity (*p* = 0.032), daily amount of medications (*p* = 0.038) and the age of severe mental illness onset (*p* < 0.001), were also significantly different among the groups. Substance use disorder patients with comorbid schizophrenia were younger than patients with comorbid major depressive disorder and have fewer years of schooling than those with comorbid bipolar disorder. A greater proportion of patients with co-existing bipolar disorder were receiving a disability pension, these patients also had the lowest rate of medical disease comorbidity. Patients with comorbid major depressive disorder were the oldest and a higher proportion was employed compared to patients in the other two groups. The group with co-existing major depressive disorder also had the highest rate of medical disease comorbidity and the oldest age of severe mental illness onset.

**Table 1:** Sociodemographic and clinical characteristics for substance use disorder patients with comorbid severe mental illness

	Substance use disorder groups with comorbid severe mental illness				
	Schizophrenia (*n* = 37)	Bipolar disorder (*n* = 34)	Major depressive disorder (*n* = 36)	ANOVA and Chi-Square	Post-hoc contrasts
Sociodemographic characteristics					
Age *(yr)*	37.78 ± 8.03	38.56 ± 8.85	42.97 ± 6.54	*F*_(2105)_ = 4.581*	0.775 (schizophrenia = bipolar disorder) 5.188* (schizophrenia > major depressive disorder) 0.413 (schizophrenia = major depressive disorder)
Living situation				*χ* ^*2*^_(2)_ = 0.496	
Living alone	25%	23.5%	30.6%		
Married or co-habiting	75%	76.5%%	69.4%		
Years of schooling	10.60 ± 2.40	12.42 ± 3.75	11.42 ± 2.44	*F*_(2105)_ = 3.344*	1.824*(schizophrenia < bipolar disorder) 0.817 (schizophrenia = major depressive disorder) 1.008 (bipolar disorder = major depressive disorder)
Economic situation				*χ* ^*2*^_(8)_ = 10.597**	
Employed Receiving unemployment benefits Disability pension No income	8.1% 21.6% 56.8% 13.5%	8.8% 14.7% 67.6% 9%	27.8% 25.0% 33.3% 13.9%		
Clinical characteristics					
Medical disease comorbidity Hypercholesterolemia Breathing system pathology Discal herniation Epilepsy HIV and/or Hepatitis (B/C) Obesity	31.4% 8.6% 5.7% 2.8% 5.7% 8.6% 0%	27.3% 9.4% 12.5% 0% 0% 2.3% 0%	55.6% 22.4% 2.8% 5.6% 5.6% 15.7% 3.5%	χ^2^_(2)_ = 6.855*	
Average number of medications per day	2.50 ± 1.13	2.24 ± 1.10	1.83 ± 1.06	*F*_(2105)_ = 3.365*	0.265 (schizophrenia = bipolar disorder) 0.667* (schizophrenia > major depressive disorder) 0.402 (bipolar disorder = major depressive disorder)
Substance use^a^					
Alcohol	72.97%	82.35%	81%	*χ* ^*2*^_(2)_ = 1.919	
Cocaine	72.97%	70.27%	64.2%	*χ* ^*2*^_(2)_ = 1.060	
Cannabis	81.08%	66.5%	52.7%	*χ* ^*2*^_(2)_ = 1.818	
Amphetamines	14.4%	18.3%	16.8%	*χ* ^*2*^_(2)_ = 1.162	
Opioids	5.8%	6%	8.4%	*χ* ^*2*^_(2)_ = 2.243	
Poly drug use	51.6%	48.4%	44.5%	*χ* ^*2*^_(2)_ = 1.237	
Number of suicide attempts	1.46 ± 1.33	1.42 ± 2.89	0.83 ± 0.97	*F*_(2105)_ = 1.217	
Mean abstinence period *(months)*	6.03 ± 3.61	6.18 ± 3.49	5.44 ± 2.51	*F*_(2, 105)_ = 0.505	
Number of relapses in the last year	1.94 ± 1.95	1.94 ± 2.87	1.63 ± 1.80	*F*_(2105)_ = 0.222	
Age of SMI onset *(yr)*	23.32 ± 5.80	26.29 ± 9.27	32.06 ± 8.75	*F*_(2105*)*_ *=* 10.383***	3.580 (schizophrenia = bipolar disorder) 8.732*** (schizophrenia < major depressive disorder) 5.152* (bipolar disorder < major depressive disorder)
Age of SUD onset (*yr)*	17.15 ± 4.70	19.20 ± 8.60	18.57 ± 7.55	*F*_(2105)_ = 0.633	
Duration of the SMI *(yr)*	13.58 ± 8.09	12.51 ± 8.52	10.91 ± 9.04	*F*_(2105)_ = 1.177	
Duration of the SUD *(yr)*	19.76 ± 8.18	19.56 ± 9.82	24.57 ± 9.41	*F*_(2105)_ = 3.254	

^a^Patients were using more than one substance

*p < 0.05; **p ≤ 0.01; ***p ≤ 0.001

**Table 2:** Comparison of clinical measures among men in treatment for substance use disorder patients by type of severe mental illness

Clinical measures	Substance use disorder groups with comorbid severe mental illness				
	Schizophrenia (*n* = 37)		Bipolar disorder (*n* = 34)	Major depressive disorder (*n* = 36)	ANOVA
DAST-20 total score	12.06 ± 0.42		11.20 ± 1.22	14.13 ± 0.65	*F*_*(*2105)_ = 3.331
PANSS	Direct scores	Percentile scores			
Positive symptoms	10.63 ± 1.21	5			
Negative symptoms	13.32 ± 1.65	15			
Composite Score	−2.69 ± 3.97	40			
General Psychopathology	28.72 ± 2.80	10			
HDRS total score			7.73 ± 0.60	12.18 ± 0.91	*F*_(1,68)_ = 6.315*
YMRS total score			2.08 ± 0.57		

DAST-20: Drug Abuse Screening Test; PANSS: Positive and Negative Syndrome Scale: HDRS: Hamilton Depression Rating Scale; YMRS: Young Mania Rating Scale

*p < 0.05; **p ≤ 0.01; ***p ≤ 0.001

All participants showed substantial severity of addiction (DAST-20) with no differences observed among the groups. Patients with comorbid schizophrenia showed low percentile scores in each dimension of the PANSS. For patients with comorbid major depressive disorder, we observed a higher depressive symptomatology than for patients with comorbid bipolar disorder (*p* = 0.002), who were euthymic according to the YMRS score.

### Health-related quality of life

Mean scores in all the primary SF-36 dimensions for the three groups are shown in Table 3. Differences among the groups were found in two primary SF-36 dimensions: Physical Functioning (*p* = 0.002) and Vitality (*p* = 0.003); while only the secondary Physical Composite Scale showed differences among the three groups (*p* = 0.001).

**Table 3:** Comparison of SF-36 dimension scores among men in treatment for substance use disorder by type of severe mental illness

Substance use disorder groups with comorbid severe mental illness						
SF-36 dimensions	Schizophrenia (*n* = 37)	Bipolar disorder (*n* = 34)	Major depressive disorder (*n* = 36)	F	Partial Eta Square	Post-hoc contrasts
Physical Functioning	80.13 ± 3.27	94.26 ± 1.93	81.97 ± 3.11	6.836**	0.117	14.484*** (schizophrenia < bipolar disorder) 4.209 (schizophrenia = major depressive disorder) 10.276* (bipolar disorder > major depressive disorder)
Social Functioning	67.92 ± 5.06	70.65 ± 5.24	71.27 ± 3.68	0.179	0.003	
Role-Physical	71.53 ± 5.91	87.55 ± 4.66	69.68 ± 5.78	2.877	0.053	
Role-Emotional	62.60 ± 6.91	58.57 ± 3.82	42.61 ± 6.58	2.293	0.43	
Mental Health	50.15 ± 3.25	51.01 ± 3.82	53.78 ± 2.93	0.251	0.005	
Vitality	41.6 ± 2.80	55.68 ± 3.66	53.63 ± 2.92	6.044**	0.105	14.148** (schizophrenia < bipolar disorder) 12.597* (schizophrenia < major depressive disorder) 1.552 (bipolar disorder = major depressive disorder)
Bodily Pain	76.56 ± 4.59	76.68 ± 4.70	68.57 ± 4.33	0.860	0.16	
General Health Perception	57.69 ± 2.95	62.05 ± 4.01	56.69 ± 3.26	0.565	0.011	
Health Changes	61.36 ± 3.94	69.85 ± 5.46	73.61 ± 4.45	1.694	0.032	
Physical Composite Scale	51.06 ± 1.41	60.40 ± 2.17	51.99 ± 1.87	7.464***	0.127	9.458*** (schizophrenia < bipolar disorder) 1.766 (schizophrenia = major depressive disorder) 7.692* (bipolar disorder > major depressive disorder)
Mental Composite Scale	37.58 ± 1.79	39.36 ± 3.42	38.61 ± 2.00	0.172	0.003	

SF-36: Short Form Health Survey

*p < 0.05; **p ≤ 0.01; ***p ≤ 0.001

Post-hoc analyses revealed poorer Physical Functioning for patients with substance use disorder and co-existing schizophrenia (*p* = 0.001; Cohen’s d = 0.687) and with co-existing major depressive disorder (*p* = 0.044; Cohen’s d = 0.797) compared to patients with comorbid bipolar disorder, and lower Vitality for patients with comorbid schizophrenia than for patients with comorbid bipolar disorder (*p* = 0.006; Cohen’s d = 0.727) and major depressive disorder (*p* = 0.020; Cohen’s d = 0.695).

Moreover, the Physical Composite Summary Scale showed lower scores for patients with co-existing schizophrenia (*p* = 0.001; Cohen’s d = 0.863) and major depressive disorder (*p* = 0.014; Cohen’s d = 0.703) than patients with co-existing bipolar disorder. No significant differences were observed among the groups in the other SF-36 dimensions.

**Fig. 2:**
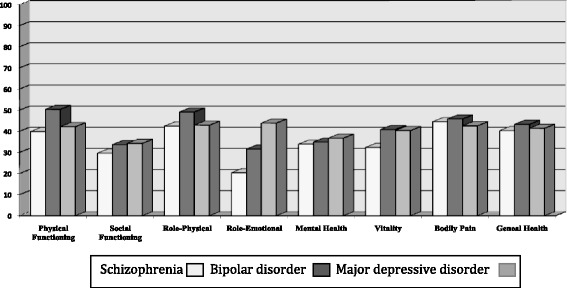
T scores (mean = 50; SD = 10) for the three groups of patients with substance use disorder and severe mental illness in the 36-Item Short Form Health Survey (SF-36) according to Spanish population norms

Figure 2 compares means for the SF-36 dimensions for the three substance use disorder groups with comorbid severe mental illness and the Spanish population norms [33]. Mean scores for the three groups were lower (−1 or −2 *SD*) than the Spanish normative data in all the primary SF-36 dimensions except for the group with comorbid bipolar disorder in Physical Functioning and Role-Physical. The lowest scores were observed in patients with comorbid schizophrenia for Social Functioning, Role-Emotional and Vitality.

### Factors contributing to health-related quality of life

Table 4 shows all linear regression models (only independent variables *p* < 0.05 were included in the model) for each of the HRQoL dimensions measured by the SF-36. Clinical variables, such as age of substance use disorder and severe mental illness onset, average number of medications per day, mean abstinence period, duration of the substance use disorder and severe mental illness, did not correlate significantly with HRQoL dimensions in any of the groups. The main results from the regression analyses indicated several clinical variables related to SF-36 dimensions by type of co-existing severe mental illness.

**Table 4:** Clinical variables and the SF-36 for substance use disorder patients with severe mental illness

	Substance use disorder groups with comorbid severe mental illness						Major depressive disorder (*n* = 36)		
	Schizophrenia (*n* = 37)			Bipolar disorder (*n* = 34)					
SF-36 Dimension	Adjusted R^*2*^	IV	ß standardized	Adjusted R^*2*^	IV	ß standardized	Adjusted R^*2*^	IV	ß standardized
Physical Functioning							0.326	Medical disease comorbidity	−0.588***
Social Functioning	0.408	Positive symptoms (PANSS)	−0.650**						
		Medical disease comorbidity	−0.432*						
Role-Physical	0.140	Suicide attempts	−0.407*	0.193	Suicide attempts	−0.467**	0.209	Medical disease comorbidity	−0.482**
Role-Emotional				0.476	Medical disease comorbidity	−0.718**			
Mental Health	0.347	Medical disease comorbidity	−0.638*	0.427	Severity of substance use disorder (DAST-20)	−0.689**	0.482	Depressive symptoms (HDRS)	−0.719**
Vitality	0.448	Drug relapses	−0.669*						
Bodily Pain	0.247	PANSS composite score	−0.532*				0.177	Depressive symptoms (HDRS)	−0.469*
General Health							0.222	Medical disease comorbidity	−0.494**

SF-36: Short Form Health Survey

In all cases Tolerance values were higher than 0.954 and Variance Inflation Factor were higher than 1

*p < 0.05, **p < 0.01; ***p < 0.001

For the comorbid schizophrenia group, positive symptoms (PANSS) (*p =* 0.002) and medical disease comorbidity (*p =* 0.030) were negatively related to Social Functioning and explained 40.8% of the variance (*F*_(1,35)_ = 7.560; *p* = 0.004). Suicide attempts were negatively linked to Role-Physical accounting for 14% of the variance (*F*_(1,35)_ = 6.556; *p* = 0.015) and Mental Health was negatively related to medical disease comorbidity explaining 34.7% of the variance (*F*_(1,35)_ = 6.851; *p* = 0.020).Vitality was negatively linked to drug relapses which accounted for 44.8%of its variance (*F*_(1,35)_ = 8.112; *p* = 0.017), while Bodily Pain was negatively related to PANSS composite score scale accounting for 24.7% of the variance (*F*_(1,35)_ = 7.900; *p* = 0.011). The severity of the substance use disorder and suicide attempts were not retained in the regression models for the comorbid schizophrenia group.

For patients with co-existing bipolar disorder, suicide attempts were negatively associated to Role-Physical which explained 19.3% of the variance (*F*_(1,32)_ = 8.665; *p* = 0.006) and medical disease comorbidity was negatively linked to Role-Emotional explaining 47.6% of the variance (*F*_(1,32)_ = 12.791; *p* = 0.004). Finally, severity of addiction (DAST-20) was negatively linked to Mental Health and accounted for 42.7% of the variance (*F*_(1,32)_ = 9.960; *p* = 0.009). For patients with comorbid bipolar disorder clinical variables including manic and depressive symptoms were not retained in the regression models.

For the comorbid major depressive disorder group, medical disease comorbidity was negatively related to Physical Functioning, accounting for 32.6% of the variance (*F*_(1,34)_ = 17.964; *p* < 0.001), and to Role-Physical explaining the 20.9% (*F*_(1,34)_ = 10.274; *p* = 0.003). Moreover, depressive symptoms (HDRS) were negatively associated with Mental Health accounting for 48.2% of the variance (*F*_(1,34)_ = 14.962; *p* = 0.002) and medical disease comorbidity was negatively associated to General Health and accounted for 22.2% of its variance (*F*_(1,32)_ = 10.995; *p* = 0.002). For patients with comorbid major depressive disorder, suicide attempts and severity of the substance use disorder were not retained in the regression analyses.

With regards to the SF-36 secondary dimensions, clinically related factors were only observed within the comorbid major depressive disorder group with scores in the Physical Composite Scale negatively linked to medical disease comorbidity (*β* = −0.684) and explaining 44.1% of the variance (*F*_(1,34)_ = 17.561; *p* < 0.001).

## Discussion

This study explores and compares for the first time the differences in HRQoL among male patients with substance use disorder and different co-existing severe mental illness. Patients with co-existing schizophrenia and with co-existing major depressive disorder showed poorer Physical Functioning and lower scores in the Physical Composite Scale than patients with co-existing bipolar disorder, while patients with comorbid schizophrenia showed a lower Vitality than those with bipolar disorder and major depressive disorder. Therefore, our results indicate that the type of co-existing severe mental illness appears to influence some HRQoL dimensions. All patients in our sample had poorer HRQoL (especially Social Functioning, Role-Emotional and Mental Health) compared with population norms. We only observed similar scores to norms for patients with co-existing bipolar disorder in Physical Functioning and Role-Physical. Our results extend previous findings [18–22, 26–29] as some of the HRQoL dimensions were impaired for the three groups of patients in our sample when compared to population norms.

Firstly, we observed major difficulties for patients with comorbid schizophrenia and major depressive disorder as they showed poorer Physical Functioning than patients with bipolar disorder and population norms. These results extend previous findings [18–20, 27] about schizophrenia and major depressive disorder patients being more likely to experience difficulties in developing vigorous activities, lifting and carrying groceries, using stairs, and walking. Secondly, scores in Vitality showed that comorbid schizophrenia patients were more frequently tired and worn out than those patients with bipolar disorder, major depressive disorder, and population norms. Contrary to previous findings, no relation was found between tiredness and daily amount of medications [20] for patients with comorbid schizophrenia. This fact could be explained by the stable clinical situation in our sample for both substance use disorder and schizophrenia diagnoses as they were receiving maintenance doses of antipsychotics.

Lastly, only one of the secondary composite scales showed differences among groups. Patients with comorbid schizophrenia and major depressive disorder showed lower scores in the Physical Composite Summary Scale than patients with comorbid bipolar disorder; this fact could be explained by their limitations in physical activities (poorer Physical Functioning), and for patients with comorbid major depressive disorder, by the presence of medical disease comorbidity. No clinical variables were related to the Physical Composite Summary Scale for patients with co-existing schizophrenia or bipolar disorder. However, and in line with previous studies [28, 29], medical disease comorbidity was negatively related to the Physical Composite Summary Scale for patients with co-existing major depressive disorder. Thus, medical disease comorbidity in patients with major depressive disorder could be a confounding factor, potentially related to the older age of these patients, which future studies should take into account and explore in detail. Nevertheless, medical comorbidities in patients with substance use disorder and major depressive disorder could be especially taken into account during their substance use disorder treatment as previous research has shown that targeting somatic comorbidities may play an important role in improving HRQoL [45].

Likewise, we observed that different clinically related factors were contributing to different HRQoL depending on the type of severe mental illness. For patients with co-existing schizophrenia positive symptoms and medical disease comorbidity were related to poorer Social Functioning, suicide attempts were associated to a worse Role-Physical, the presence of medical disease comorbidity was linked to worse Mental Health, drug relapses were negatively to a lower Vitality and the predominance of negative symptoms was associated with Bodily Pain. For patients with co-existing bipolar disorder, a worse Role-Physical was associated with suicide attempts, medical disease comorbidity was linked to worse Role-Emotional and a higher severity of the substance use disorder was linked to a worse Mental Health. These results for patients with comorbid bipolar disorder extend previous data explaining the association among suicide attempts [24], medical disease comorbidity [23] and HRQoL impairments for patients with bipolar disorder. Regarding patients in the comorbid major depressive disorder group, poorer Physical Functioning, and poorer Role-Physical were associated with the presence of medical disease comorbidity. In line with previous data [28, 29] higher depressive symptoms were associated with a worse Mental Health while medical disease comorbidity was linked to a worse General Health.

The differences we observed among groups have clinical implications and highlight the need to adjust treatment approaches to address such difficulties especially among substance use disorder male patients with comorbid schizophrenia and with comorbid major depressive disorder. For instance, substance use disorder treatments could include self-directed exercise, as it is an effective way to improve Physical Functioning [46], and positive compensation strategies to enhance patients’ performance capacity [47]; as these interventions improve physical and general wellbeing [48]. To potentially improve Vitality, especially in patients with substance use disorder and co-existing schizophrenia fatigue and low energy levels could be addressed with interventions that use motivational strategies to engage patients in exercise emphasizing that working-out may ameliorate these symptoms too. Similarly, treatment could include strategies to manage other Vitality related factors such as sleep, since previous studies found that better sleep quality improves HRQoL and increases energy levels [49].

Furthermore, consistent with previous observations about different substance use disorder and/or severe mental illnesses [9, 19–22], our findings showed impaired HRQoL dimensions (especially Social Functioning, Role-Emotional, and Mental Health) in the three groups compared to population norms; except for Physical Functioning and Role-Physical in comorbid bipolar disorder patients. A normal Physical Functioning and Role-Physical for patients with bipolar disorder according to norms could be potentially explained by patients in this group being euthymic and having the lowest rates for medical disease comorbidity. In this sense, patients with substance use disorder and co-existing bipolar disorder had better clinical characteristics than those with schizophrenia and with major depressive disorder. Dually diagnosed male patients in our sample experienced frequent limitations with normal social activities due to emotional problems (poorer Social Functioning) and this was more notable for those patients with comorbid schizophrenia and related to their positive symptomatology. A potential explanation of these results could be the burden of severe mental illness and the impact of drug dependence in the relationship between the individual and its social network; patients in our sample were in a clinically stable but not completely asymptomatic during our assessment. Patients from all groups, and especially those with co-existing major depressive disorder, had difficulties with work or other daily activities as a result of emotional problems (poorer Role-Emotional); these difficulties were positively associated with suicide attempts for patients with comorbid schizophrenia. All patients frequently reported feelings of nervousness and depression (worse Mental Health) and this was especially associated with medical disease comorbidity for patients with schizophrenia, the severity of addiction for bipolar disorder, and depressive symptomatology for major depressive disorder.

Our results support the importance of an integrated recovery model [12, 15, 30] that does not only consider abstinence as the main therapeutic goal for patients with substance use disorder and psychiatric comorbidity. For instance, treatments for substance use disorder male patients with co-existing schizophrenia should include strategies to increase social activities (e.g. group activities for a better Social Functioning); while interventions targeted at male patients with co-exiting major depressive disorder should include techniques to cope with depressive symptoms (e.g. behavioral activation to improve Role-Emotional). Besides, for male patients with substance use disorder and a co-existing schizophrenia, bipolar disorder or major depressive disorder, treatments should consider strategies to manage emotional problems (e.g. coping strategies focused on emotion) and reduce anxiety (e.g. relaxation techniques) as they had poorer Mental Health than population norms. Such strategies could improve patients´ HRQoL and therefore, enhance their functioning and treatment adherence [15, 17].

### Limitations

The present study has some potential limitations. The cross-sectional design only allowed us to explore the weight of each psychiatric condition, as we could not investigate causal relationships between substance use disorder, comorbid severe mental illness and HRQoL. The sample size was small and only included substance use disorder male patients with schizophrenia, bipolar disorder, and major depressive disorder who were receiving treatment for addiction. Therefore, our results can only be extended to male patients in treatment for substance use. In addition, data were based on partially retrospective self-reported information that may have been subject to recall bias. Further studies should include females and compare results by gender. Adequately powered samples using a longitudinal design could assess the impact of treatment on HRQoL.

Despite these limitations, our study supports the importance of assessing and managing HRQoL in male patients with substance use disorder and severe mental illness as they showed poorer HRQoL. Therefore, substance use disorder treatments should be matched to different types of severe mental illness, as poorer HRQoL found in some dimensions differed according to the co-existing severe mental illness. Hence, future studies examining HRQoL in patients with substance use disorder should consider comorbidity as a mediating factor as it is linked to both substance use disorder and psychiatric variables [14, 25] and is one of the recommended outcome measures that could help to improve clinical care and design tailored treatment approaches [15, 16].

## Conclusions

In summary, male patients with substance use disorder showed differences in HRQoL depending on the type of co-existing severe mental illness. Patients with substance use disorder and comorbid schizophrenia and with comorbid major depressive disorder showed poorer Physical Functioning than those with comorbid bipolar disorder, while those with comorbid schizophrenia showed the lowest Vitality. Different clinical variables (e.g. medical disease comorbidity, severity of addiction, drug relapses and psychiatric symptoms) were related to different HRQoL dimensions depending on the co-existing severe mental illness diagnosis. In addition, compared to Spanish population norms, Social Functioning, Role-Emotional, and Mental Health dimensions were significantly lower in the three groups. We only observed scores similar to norms for patients with substance use disorder and comorbid bipolar disorder in Physical-Functioning and Role-Physical. Therefore, among male patients with substance use disorder, the type of severe mental illness could influence some HRQoL dimensions as well as their clinically related variables.

## Abbreviations

ANOVA: Analysis of Variance
BD: Bipolar Disorder
DAST-20: Drug Abuse Screening Test
HDRS: Hamilton Depression Rating Scale
HRQoL: Health-Related Quality of Life
MANCOVA: Multiple Analysis of Covariance
MDD: Major Depressive Disorder
PANSS: Positive and Negative Syndrome Scale
SCID-I: Structural Clinical Interview for DSM-IV-TR Axis I Disorders
YMRS: Young Mania Rating Scale
